# Different natural languages, equal importance

**DOI:** 10.1016/j.patter.2023.100821

**Published:** 2023-08-11

**Authors:** Wanying Wang

**Affiliations:** Scientific editor, *Patterns*

With the wide applications of large language models (LLMs), natural language processing (NLP) is no longer just jargon for many people. NLP is a branch of artificial intelligence (AI) that enables computers to understand human languages. We are already reaping the benefits of NLP in our daily lives, such as translation, spam detection, and chatbots, but at the same time, there is reason to worry about the consequences of the rapid development of these technologies. Apart from fears of humans being replaced by AIs, one NLP-specific concern is that the underrepresentation of non-English languages in NLP research and models could undermine the benefits for speakers of other languages.

There are estimated to be more than 7,000 actively spoken languages in the world (Ethnologue | Languages of the world), each of which is vital to communication within their speech communities. More importantly, diverse cultures are inherited and represented by different languages. NLP tools and research are biased, however, by the asymmetry in language resources. It is "natural” that English has become a mainstream language in different NLP scenarios. The number of speakers and the number of researchers working in and on a specific language heavily determine the provision of language resources, which leads to disparities that are evident across NLP publications (D. Blasi et al., 2022, Assoc. Comput. Linguist., http://dx.doi.org/10.18653/v1/2022.acl-long.376). To help dispel the notion that English should always be considered the default in NLP, Professor Emily Bender proposed in 2011 that researchers should “always name the language you’re working on,” which has become known as the “Bender rule” (Bender, 2011. Linguist. Issues Lang. Technol. 6., 1–28, https://doi.org/10.33011/lilt.v6i.1239).

In the last decade, linguistics and NLP research communities have been going past this minimum standard and making active efforts to promote recognition of and research on non-English languages. Since 2020, the AfricaNLP workshops have run successfully as part of the International Conference on Learning Representations. Recent annual meetings of the Association for Computational Linguistics (ACL) also organized a track titled “Language Diversity: From Low-Resource to Endangered Languages” and a tutorial titled “Everything you need to know about Multilingual LLMs: Towards fair, performant, and reliable models for languages of the world” to encourage the diversity of languages.

The awareness of language bias is spreading from NLP research to wider scientific communities. More language-diverse NLP tools could potentially improve the viability of research projects run by researchers who speak different languages. In an issue of *Cell* earlier this year, three non-native-English-speaking scientists called on “Enhancing translation of science into non-English languages” (Sharma et al., 2023, Cell 186, 1097–1098, https://doi.org/10.1016/j.cell.2023.01.036), in which they presented approaches enabling better access to scientific content in non-English speaking countries. As a matter of course, if NLP models are not constrained by language differences, they will better assist in translating scientific findings across languages and progressively accelerate scientific research.

In this issue of *Patterns*, a group of researchers from the Masakhane Research Foundation identify core stakeholders involved in African NLP processes and explore their motivations, focuses, and challenges (Siminyu et al., 2023, Patterns *4*, 100820, https://doi.org/10.1016/j.patter.2023.100820). They highlight four main outcomes. First, African languages, just like other languages, represent the cultural identities that are crucial for African identity and societal participation. Second, collaborative support for African content creation is needed, from creating African terminology to building basic language technologies, implementing language policies, and enlarging the market for African language skills. Third, from the perspective of creating African language technologies, the interviewees point out the importance of interdisciplinary training opportunities and collaboration with linguists. Finally, concerns about data governance for language data were captured in terms of data collection, data curation, and the openness of datasets. At the end of this paper, a detailed set of recommendations are put forward that could benefit the African language ecosystem. It is not difficult to appreciate that the challenges faced by African languages are common in other low-resource languages.

We therefore genuinely hope that more projects like Masakhane will contribute to the development of non-English NLP applications and help reduce the pervasive English biases in AI technologies. As scientific editors, we understand the central role of academic publishing in promoting and advancing scientific findings, and we must acknowledge, at the same time, that it plays a role in propagating pro-English bias. From my personal perspective, I am a native Mandarin speaker, the second most spoken language in the world, but I use English to communicate with our peer reviewers, authors, and readers. For non-native English speakers hoping to be among these three groups, cutting-edge expertise in a relevant research area is not enough; English language skills sufficient for advanced scientific communication are also required. While LLM-empowered translation or grammar correction tools may help non-native English speakers write or read English content, these tools come with important caveats, and users should take care to comply with journal policies on their use (see our April editorial).

Nonetheless, we feel that these are challenges that can and must be faced. We hereby invite researchers with compelling new advances in non-English NLP to submit their work to the journal for consideration and possible publication. This is not limited just to groundbreaking technical NLP advances and solutions but also open to research papers that use these or similar technologies to push language and domain boundaries. We commit ourselves to seeking creative ways to peer review each such submission in a timely manner that is rigorous, respectful, and consistent with the journal’s high standards. Authors are encouraged to reach out to us beforehand with a cover letter and a brief description if they would like advice before a formal submission.

